# Exploiting Ultra-Wideband Channel Impulse Responses for Device-Free Localization

**DOI:** 10.3390/s22166255

**Published:** 2022-08-20

**Authors:** Marco Cimdins, Sven Ole Schmidt, Peter Bartmann, Horst Hellbrück

**Affiliations:** Department of Electrical Engineering and Computer Science, Technische Hochschule Lübeck, Mönkhofer Weg 239, 23562 Lübeck, Germany

**Keywords:** device-free localization, channel impulse response, ultra-wideband, radio tomographic imaging, multi-static radar, multipath components, multipath-assisted

## Abstract

In radio-frequency (RF)-based device-free localization (DFL), the number of sensors acting as RF transmitters and receivers is crucial for accuracy and system costs. Two promising approaches for DFL have been identified in the past: radio tomographic imaging (RTI) and multi-static radar (MSR). RTI in its basic version requires many sensors for high accuracy, which increases the cost. In this paper, we show how RTI benefits from multipath propagation. By evaluating the direct and echo paths, we increase the coverage of the target area, and by utilizing UWB signals, the RTI system is less susceptible to multipath propagation. MSR maps reflections that occur within the target area to reflectors such as persons or other objects. MSR does not require that the person is located near a signal path. Both suggested methods exploit ultra-wideband (UWB) channel impulse response (CIR) measurements. CIR measurements and the modeling of multipath effects either increase the accuracy or reduce the required number of sensors for localization with RTI. We created a test setup and measure UWB CIRs at different positions with a commercially available off-the-shelf UWB radio chip, the Decawave DW1000. We compare the localization results of RTI, multipath-assisted (MA)-RTI, and MSR and investigate a combined approach. We show that RTI is improved by the analysis of multipath propagation; furthermore, MA-RTI results in a better performance compared to MSR: with 50% of all cases, the localization error is better than 0.82 m and in 80% of all cases 1.34 m. The combined approach results in the best localization result with 0.64 m in 50% of all cases.

## 1. Introduction

Radio-frequency (RF)-based device-free localization (DFL) systems monitor the target area by measuring changes in the radio channel. Persons or other objects can be detected [1], counted [2], and localized. An advantage of RF-based DFL systems is that channel measurements such as the received signal strength indicator [3], channel state information [4], or channel impulse responses (CIRs) are available for regular RF transmissions. RF-based DFL systems exploit the radio channel measurements as sensor readings.

We distinguish between wideband and narrowband radio technologies, where wideband or even ultra-wideband (UWB) technologies are evolving. In narrowband DFL systems, multipath propagation results in constructive and destructive interference and prevails in that changes in the channel state can be mapped reliably to specific regions [5]. To overcome this drawback, the authors propose to combine multiple narrowband channels [6,7,8], which is a development comparable to a wideband system.

The focus of the paper is on multipath methods for UWB DFL systems. The UWB radio chip Decawave DW1000 provides complex-valued CIR measurements that include information about the direct path (sometimes called line-of-sight path) and echo paths. These CIR measurements vary in the magnitude and phase of multipath components (MPCs) when persons move in the direct or any echo path. UWB enables the extraction of MPCs and, respectively, mapping those MPCs to the target area. The high bandwidth, in this paper, approximately 500 MHz, allows distinguishing of MPCs when they are 1 ns apart from each other in the time domain. This enables distinguishing whether a person is in the proximity of any signal path.

Multipath localization has been the focus of research for many years, e.g., many tag-based localization systems, i.e., systems that locate tags by calculating, e.g., the time-of-flight between the tag and anchors, already exploit multipath to reduce the number of required sensors [9,10,11]. Different from DFL systems, tag-based localization systems see persons as obstacles that obstruct the line-of-sight path and, therefore, decrease the localization accuracy [12].

**Figure 1:**
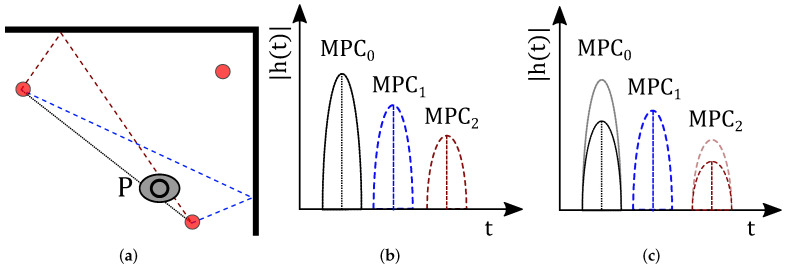
Principle of MA-RTI for UWB CIR measurements. (**a**) Concept of MA-RTI. (**b**) CIR when the target area is empty; idle case. (**c**) CIR when the person is located within the target area.

**Figure 2:**
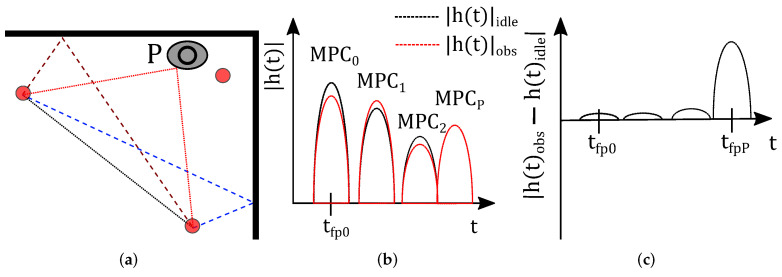
Principle of MSR for UWB CIR measurements. (**a**) Concept of MSR. (**b**) CIR measurement with idle and non-idle target area. (**c**) CIR difference.

In this paper, we investigate two DFL methods that we will enhance to cope with and benefit from multipath propagation: The first method is radio tomographic imaging (RTI). RTI maps the attenuation of radio links into a heat map and, thus, localizes a person, where a radio link is the radio communication between transmitter and receiver. As a main contribution, we enhance RTI to benefit from multipath propagation and, thereby, increase the likelihood of detecting a person in either the direct path or in echo paths resulting from reflections on walls. The second method, multi-static radar (MSR), exploits the fact that persons create reflections that change the CIR. MSR approaches map additional reflections within the target area, thus localizing the person. Figure 1 and Figure 2 show the principle of how a person affects the channel measurement of both methods. Both methods will be described in detail in Section 3. The red dots are sensor nodes that communicate via UWB radio technology. The black dotted line represents the direct path between each sensor pair. What is new in this work is that we consider also the echo paths (dashed lines), which are the first-order multipath components, i.e., RF paths based on a single bounce reflection on the walls depicted as the bold black lines in Figure 1.

In the following, we describe how a person affects the MPCs that are extracted from the UWB CIR measurement: In Figure 1a, the person *P* located at position $r_{P}$ affects the direct path between two sensors (${\mathrm{MPC}}_{0}$) and the red dashed echo path (${\mathrm{MPC}}_{2}$). When the target area is vacant, i.e., free of additional persons, we measure an idle CIR and determine the magnitude of the MPCs (see Figure 1b). We will later use this idle case to subtract the background and identify changes in the MPCs due to the position of the person. Changes in the respective MPCs, in comparison to the idle CIR, shown in Figure 1c, limit the possible locations of the person. To find the position of the person, radio tomographic imaging (RTI) [6,7,13,14] or fingerprinting (FP) algorithms [15,16,17] are used. RTI algorithms map changes in the signal paths and display them in a heat map. In this work, we enhance RTI both to use the direct and echo paths of the UWB signal and describe the mapping of changes in the MPCs into a heat map. Fingerprinting algorithms compare the distance of the channel measurements prerecorded at reference positions with measured observations to determine the position of the person. Other possibilities for localization are geometric approaches [18] and compressed sensing [19,20].

MSR approaches utilize changes within the UWB CIR (see Figure 2). In Figure 2a, the person stands in the middle of the target area. Here, he/she does not block any signal path of the sensor pair. In the CIR, we will see a new MPC by the reflection at the person. Different from RTI, MSR does not depend on the blocking of the signal paths. After background subtraction, it detects and exploits significant changes in the channel measurement.

Figure 2b shows the CIR measurement while the target area is vacant (black dotted CIR). A person that stands in the middle of the target area (see Figure 2a) creates an additional MPC named ${\mathrm{MPC}}_{P}$, shown in Figure 2b as the red dotted line. Subtracting the CIR of the non-idle case from the CIR of the idle case shows differences due to the person within the target area; this step is called background subtraction. MSR now maps the time delay of the newly occurring MPC, which limits the possible position of the person within the target area.

In this work, we address multipath propagation for RTI and MSR. Furthermore, we propose to apply both DFL methods to the same set of UWB CIR measurements. We compare RTI, MA-RTI, and MSR within a real measurement setup and propose a novel scheme that combines MA-RTI and MSR to combine their advantages. We perform UWB CIR measurements with a commercial off-the-shelf (COTS) radio chip, the Decawave DW1000. Different from the Bayes methods such as Kalman or particle filters [21,22], MA-RTI and MSR create intuitive heat maps of the target area. A deeper understanding of the advantages of the effect on the CIR will also lead to better filters in the future. To the best of our knowledge, the combination and comparison of those DFL methods from real CIR measurements have not been performed before.

Our contributions are as follows:

- We demonstrate how to exploit information from UWB CIR measurements to derive the position of a person within a target area with different DFL methods, namely RTI, MA-RTI, MSR, and a combination of MA-RTI and MSR.
- We enhance RTI methods to benefit from multipath to reduce the number of required sensors and make RTI more robust against multipath propagation.
- We test MSR under real conditions and propose to defeat multipath propagation by the so-called background subtraction.
- We compare RTI, MA-RTI, and MSR and combine MA-RTI and MSR within a test setup.

The rest of the paper is structured as follows: Section 2 presents the related work of DFL systems and multipath models. In Section 3, we propose the idea of the solution, including a novel multipath-assisted RTI method, an MSR method, and their combination. Section 4 shows the details of the implementation, such as the measurement hardware, firmware, measurement setup, and processing of the measurements. We evaluate the DFL methods and their combination in Section 5. Finally, we conclude our work and give an outlook for future work in Section 6.

## 2. Related Work

In this section, we provide a brief overview of the related field in the research on DFL systems. Youssef et al. proposed the first DFL system based on fingerprinting in [15], enabling research in this field. A few years later, Wilson and Patwari proposed RTI, an approach that avoids the extensive training phase of a fingerprinting system, but requires many sensors [13].

One major challenge of DFL systems, especially in indoor environments, is multipath propagation, which leads to the constructive and destructive interference of the received signal [5]. When multipath propagation affects the channel measurement more strongly than the person, the performance of the DFL system degrades.

Although modeling the influence of a person and multipath propagation on the received signal is possible [5], such approaches require exact knowledge of the sensor and multipath positions, which is impractical in real deployments. Therefore, different authors proposed to combine several RF channels for DFL and chose the radio links that are least affected by multipath or combined the results [6,7]. Schröder and Wolf provide another possibility to avoid narrowband channel measurements in [23]. They swept through the 2.4 GHz ISM band and recorded the measured phase between two IEEE 802.15.4 nodes.

UWB radar sensor networks aim to localize persons within a target area, e.g., mono-static UWB sensor radar networks are proposed in [24,25]. Mono-static UWB radar sensor networks require that the same sensor node be able to receive the transmitted UWB pulses, e.g., with a second receive antenna. Multi-static UWB radar sensor networks are proposed in [26,27,28]; there, one sensor node transmits UWB pulses, and at least one different node acts as the receiver. Those approaches have in common that they calculate the time-of-arrival upon receipt of the signal. With this information, tracking is performed, e.g., with the help of particle filters.

Different from our MSR approach, the authors acquired the time-of-arrival information upon receipt of the UWB pulses. Our solution is based on a COTS UWB radio chip, the Decawave DW1000, which enables the extraction of CIR measurements. The extracted CIR is processed, and information about the person is extracted and compared with several DFL methods.

UWB CIRs enable the exploitation of multipath components and can be measured using COTS low-cost UWB radio chips. On the one hand, UWB signals reduce the effect of multipath propagation for DFL systems. On the other hand, this helps reduce the number of required sensors for localization. In [29], Moschevikin et al. described a method to carefully align CIR measurements of the Decawave DW1000, enabling pre-processing of the CIR. To model changes in the magnitude and phase of the UWB CIR due to the position of a person, we refer to [30]. In previous work, we proposed to exploit multipath propagation, as well as the magnitude and phase of UWB CIRs to increase the accuracy of DFL systems and to reduce the number of physical sensor nodes [31,32]. However, those approaches depend on fingerprinting algorithms, which require an extensive training phase. Therefore, in this paper, we focus on methods that only require idle measurements for calibration and background subtraction.

RTI has been excessively researched for narrowband DFL systems [6,7,8,13]. To the best of our knowledge, there have been only a few works including multipath propagation in RTI systems. In narrowband DFL systems, multipath propagation typically reduces the accuracy of RTI [33]. Kim et al. created a system that includes multipath with mmWave radios [34]. In [35], Zhang et al. introduced RTI with multipath, and for that, they included angle-of-arrival measurements with RFID. In this paper, we propose multipath-assisted (MA)-RTI, which extracts MPCs from the UWB CIRs and, therefore, benefits from the echo paths of the signal.

Next to conventional DFL approaches such as fingerprinting or RTI, UWB CIR measurements enable MSR approaches. Ledergerber et al. proposed an MSR based on UWB [21]. From the variances of the UWB CIR measurements, they extracted the time delay of the new reflection and determined the position of a person with a particle filter. In [36], Li et al. used the dataset provided by Ledergerber et al. [21] to track persons. They used a convolutional neural network for time-of-flight estimation and deployed a particle filter for tracking persons. In [37], the same authors elaborated on practical concerns of MSRs for pedestrian tracking. Bocus and Piechocki used the dataset from Ledergerber et al. to implement passive tracking [38].

Another possibility to set up MSR is to draw the CIR differences in ellipses on the target area, as performed in [39] in an anechoic chamber. Later, Ninnemann et al. applied this technique for the occupancy detection of cars [40] and for detection of persons within an aircraft cabin [41]. As MSR is a promising approach, we adapted the MSR approach proposed in [39] and tested this approach under multipath conditions and with a person to be localized. Therefore, we added a background subtraction, as proposed in [40].

In [42], Hong et al. proposed a device-free angle-of-arrival estimation with UWB signals. Exploiting the angle-of-arrival measurements increases the accuracy; however, this requires multiple antennas on the receiver side. Furthermore, UWB CIR responses enable device-free activity detection, as shown in [43]. Other approaches include MPCs with Bayesian filtering to localize and track a person [22].

Recent research proved that MSR algorithms detect and track persons by variations of the UWB CIR. However, those methods rely on the dynamic variation of the CIR (e.g., the variances). Such approaches perform well in scenarios with moving persons, but fail with static persons. In this paper, we strive for two goals: First, we propose to enhance RTI by processing multipath signals that are extracted from a UWB CIR. Second, we combine MA-RTI with MSR. Both methods were extracted from the same UWB CIR. To the best of our knowledge, a comparison and combination of those DFL methods based on UWB CIRs has not been proposed before.

## 3. Approach

In the first part of this section, we describe the signal propagation including multipath components (MPCs). In the second part, we propose multipath-assisted (MA) radio tomographic imaging (RTI), a DFL method that uses MPCs for the localization of the person. We describe how RTI is applied to the direct and echo paths that are extracted from UWB CIR measurements to decrease the number of physical sensors needed. In the third part of this section, we present multi-static radar (MSR), an approach that scans the CIR for new reflections in order to detect the person.

### 3.1. Signal Propagation Including Multipath Components

In this paper, we extract and exploit MPCs from the UWB CIR. Therefore, we describe the propagation of UWB signals in this section.

A UWB pulse x(t) sent by the transmitter Tx is sensed by the receiver Rx. The pulse propagates in a direct path between Tx and Rx and additional echo paths reflected on walls and other obstacles. All signal components are received by Rx. The echos of x(t) passing the *i*-th path are received with a corresponding time delay $\tau_{i}$ and a decreased amplitude $a_{i}$. The CIR h(t) depicts the multipath propagation of all *I* signal echos between Tx and Rx:

(1) $$\begin{array}{c} h\left(t\right)=\sum_{i=0}^{I-1}a_{i}\delta_{0}\left(t-\tau_{i}\right), \end{array}$$

where $\delta_{0}\left(t\right)$ is the Dirac function.

The received signal y(t) at Rx is a superposition of all received signal echoes:

(2) y(t)=x(t)∗h(t)+n(t),

where n(t) is additive white Gaussian noise [44].

**Figure 3:**
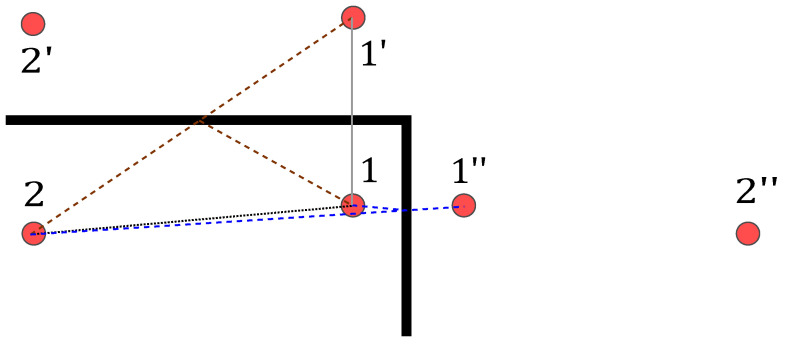
Visualization of multipath components and the position of virtual sensors.

Figure 3 shows an exemplary multipath scenario with two walls. The black dotted line between Sensor 1 and Sensor 2 is the i=0-th component, which is the direct path between the transmitter and the receiver. Additionally, we depict echo paths that are the first-order MPCs in blue and brown dashed lines. To determine the echo paths, the positions of virtual sensors $1^{\prime },2^{\prime },1^{\prime \prime }$, and $2^{\prime \prime }$ are calculated. These virtual sensor positions result from mirroring the physical sensor positions on the walls, as shown as the gray line in Figure 3 on Sensor 1, e.g., the upper reflection (brown dashed line) is calculable with respect to virtual sensor position $1^{\prime }$. The path length of the connection between 2 and $1^{\prime }$ equals the MPC’s length, and the connection’s intersection with the wall is the reflection point of the MPC. Therefore, we determine the path length and the resulting time delay $\tau_{i}$.

### 3.2. Multipath-Assisted Radio Tomographic Imaging

Radio tomographic imaging (RTI) creates a heat map from the target area. A person will mainly affect an MPC when he/she is present within the first Fresnel zone of the respective path. This usually results in blind spots or the deployment of many sensors. Therefore, RTI approaches require typically 10–20 sensors to achieve a sophisticated accuracy [6,7,8,13].

One contribution of this paper is exploiting multipath propagation for RTI and proving that this multipath-assisted (MA) approach works with only a few physical sensors.

#### 3.2.1. Including MPCs in RTI

Narrowband RTI systems rely on channel measurements of radio links and are affected by multipath propagation. UWB CIR measurements enable the extraction of multiple MPCs from the CIR. In the following, we focus on an exemplary multipath scenario including two walls, as shown in Figure 3. To exploit the MPCs for RTI, every sensor position is mirrored on each wall, resulting in additional virtual sensor positions $1^{\prime }$ and $2^{\prime }$ for the top wall and $1^{\prime \prime }$ and $2^{\prime \prime }$ for the right-hand wall. In the following, we use the permutations of the physical and virtual sensor pairs, namely $\left(1,2^{\prime }\right)$, $\left(2,1^{\prime }\right)$, $\left(1,2^{\prime \prime }\right)$, and $\left(2,1^{\prime \prime }\right)$. Together with the direct path (1,2) $\left({\mathrm{MPC}}_{0}\right)$, this results in five different MPCs for each sensor pair, increasing the conventional number of paths by four echo paths, even for this simple setup. The difference from the conventional RTI system is that the former solely exploits the direct path ${\mathrm{MPC}}_{0}$ for the localization of a person. Note: With the permutations of the physical and virtual sensor pairs, we map each echo path onto the target area. As we extract the values for an echo path from the CIR, both the incident and the reflected ray will receive the same complex value. For RTI, the measurement vector z depicts the difference between the observation and the idle case modeled in a single value. For the *i*-th MPC, $z_{i}=z\left[i\right]$ is defined as:

(3) $$z_{i}=\left|P_{\mathrm{MPC},i,\mathrm{obs}}-P_{\mathrm{MPC},i,\mathrm{idle}}\right|,$$

where $P_{\mathrm{MPC},i,\cdot }$ is the signal power of the observed and idle CIR at $\tau_{i}$, respectively. The more the person affects the MPC on the direct or echo path in comparison to an idle target area, the higher the input for MA-RTI is.

In this paper, we deploy the absolute difference for each MPC as the input for the MA-RTI system, as described by (3).

Note, many narrowband RTI systems reduce the impact of multipath propagation, by calculating other features such as the variance as the input vector $z_{i}$ [7]. Using the variance, however, inherits the problem that the feature decreases when the person stands still within the target area. Although UWB signals are also affected by multipath propagation [45], extracting MPCs from the UWB CIR reduces the problem.

#### 3.2.2. Principle

In the following, we describe the principle of MA-RTI. First, we divide the target area *A* into *J* equally sized pixels. Each pixel has an assigned attenuation value, which is subject to be estimated from a measurement. The heat map (also called attenuation image) is represented by vector v. A matrix W of size I×J assigns weights $w_{ij}$ to the heat map. As a result, z is the measurement vector of length *I*, with *I* being the number of the direct and echo paths (in our case, the MPC components of all sensors).

RTI systems are based on a simple linear model [13]:

(4) z=Wv+n,

where n is *I*-dimensional normally distributed noise.

The weight $w_{ij}$ for each path *i* and each pixel *j* is determined by the following equation [13]:

(5) $$w_{ij}=\left\{\begin{array}{cc} 1/\sqrt{d_{i}} & \mathrm{if}\;d_{ij}\left(1\right)+d_{ij}\left(2\right)<d_{i}+\lambda \\ 0 & \mathrm{otherwise} \end{array}\right.,\;$$

where $d_{ij}\left(1\right)+d_{ij}\left(2\right)$ is the distance from Sensor 1 to Sensor 2 on the *i*-th path over the center of pixel *j*, $d_{i}$ is the distance of the *i*-th path, and λ is a tuning parameter in $R^{+}$ [13].

**Figure 4:**
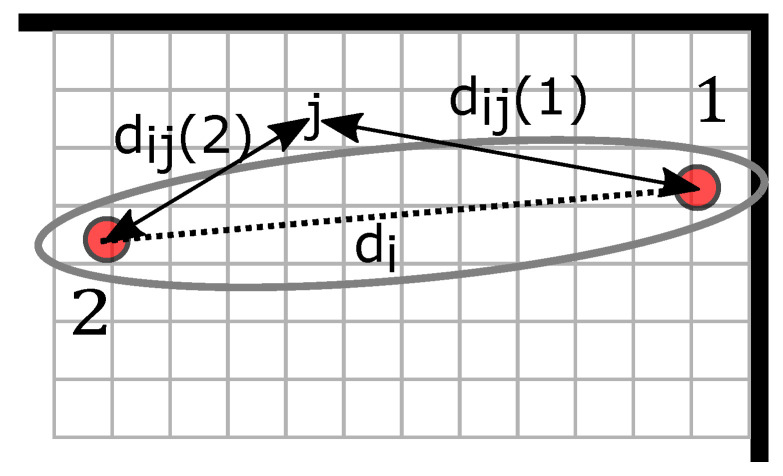
Weight function of RTI.

Figure 4 depicts the parameters. When the pixel *j* is inside an ellipse with the sensors in its foci, then the weight $1/\sqrt{\left(d_{i}\right)}$ is assigned; otherwise, the weight is 0. When a person is outside the direct or echo path, this path will not affect the RTI system. The tuning parameter λ controls the width of the ellipse.

In the following, we discuss solutions for linear equations such as (4). Such equations are typically solved by L2-minimization (6):

(6) $${\hat{v}}_{LS}=\underset{\tilde{v}\in C^{J}}{arg min}||W\tilde{v}-z{\left|\right|}_{2}^{2},$$

which leads to the well-known solution:

(7) $${\hat{v}}_{LS}=W^{†}z,$$

where $W^{†}$ is the Moore–Penrose pseudo-inverse of W. Each weight $w_{ij}$ for each path *i* and each pixel *j* is calculated as described in (5).

However, this approach leads to reliable results only if (7) is well conditioned, i.e., W has rank *J*. Unfortunately, this is not the case for an RTI system as the number of paths *I* is smaller than the number of pixels *J*. RTI is an ill-posed inverse problem; many pixels are estimated by a low number of paths, so J≫I. In addition, there are pixels that are not crossing any path; therefore, any measurement will lead to the same result, which causes an overall under-determined system [13].

Solving the equation requires regularization of the pseudo-inverse with a covariance matrix of *v* $C_{v}$ weighted by $\sigma_{J}^{-2}$. The elements of the covariance matrix are defined as [13]:

(8) $$C_{v}\left[k,l\right]=\sigma_{v}^{2}e^{-d_{kl}/\delta_{c}}$$

where $d_{kl}$ is the distance from pixel *k* to *l*, $\delta_{c}$ a space constant, and $\sigma_{v}^{2}$ is the pixel variance of the estimation error. Such an exponential covariance is a common approximation of a spatial attenuation, which is modeled as a Poisson process [13].

Including the covariance matrix in the calculation of the pseudo-inverse, (7) becomes:

(9) $$\hat{v}={\left(W^{T}W+C_{v}^{-1}\sigma_{J}^{2}\right)}^{-1}W^{T}z$$

We determine the estimated position of the person ${\hat{r}}_{P}$ by searching the maximum value in $\hat{v}$:

(10) $${\hat{r}}_{P,\mathrm{MA}-\mathrm{RTI}}=\mathrm{Pos}\left(\underset{j\in \{0\ldots J-1\}}{arg max}{\hat{v}}_{j}\right),$$

where operator Pos(j) returns the position vector of the *j*-th pixel.

Finally, we normalize $\hat{v}$ to result in ${\hat{v}}_{\mathrm{MA}-\mathrm{RTI}}$.

### 3.3. Multi-Static Radar

This section describes MSR, a method that maps the CIR to a target area. The idea of this DFL method is to detect and track persons outside of MPCs. Instead of only finding the time delay of the reflection and inserting this into a particle filter [21], we map the whole CIR difference to the target area as proposed in [39]. One contribution of this paper is adapting the MSR approach of [39] and evaluating this method in multipath conditions. Therefore, we performed measurements outside an anechoic chamber, and different from [40], we localized a person instead of a parking car.

Idea: we will both use parts of the CIR (e.g., the magnitude $P_{\mathrm{MPC}}$ of the *I* MPCs) and the complete time series. We expect that MSR works well for positions where a person does not affect a multipath component. Therefore, MSRs localize persons at positions where RTI systems provide low localization accuracy.

#### 3.3.1. Principle

An MSR detects a person with changes in the received signal compared to the idle case. The transmitter Tx and the receiver Rx are placed at known positions. Emitted signals by Tx are received at Rx on the direct path and via echo paths on persons. In a target area without walls, we expect only the direct path. When there are echo paths available, a person must be present.

**Figure 5:**
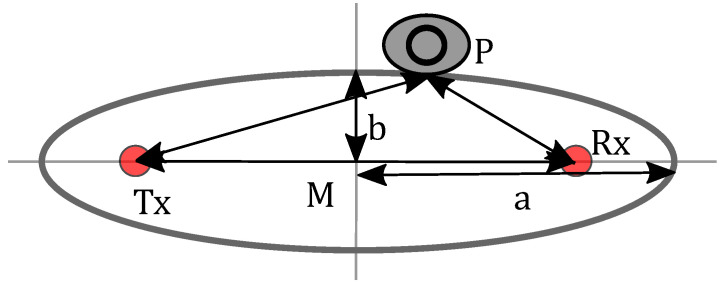
Ellipse parameters.

Figure 2c shows that the person causes a new ${\mathrm{MPC}}_{P}$ at the time $t_{\mathrm{fpP}}$. With the speed of light $c_{0}$, the distance $d_{\mathrm{fpP}}$ between the transmitter with position $r_{Tx}$ over the person at $r_{P}$ to the receiver at $r_{Rx}$ is defined by [39]:

(11) $$\begin{array}{cc} d_{\mathrm{fpP}} & =t_{\mathrm{fpP}}\cdot c_{0} \end{array}$$

(12) $$\begin{array}{cc} \; & =\left|r_{Tx}-r_{P}\right|+\left|r_{P}-r_{Rx}\right|+\epsilon_{\mathrm{fpP}}, \end{array}$$

where $\epsilon_{\mathrm{fpP}}$ models the measurement error. The solution space for $r_{P}$ draws an ellipse with Tx and Rx in the foci, as illustrated in Figure 5.

If the system utilizes *o* different sensor permutations, the solution $r_{P}$ is found by solving a non-linear system of equations [39].

(13) $$d_{i}=\left|r_{Tx,o}-r_{P}\right|+\left|r_{P}-r_{Rx,o}\right|+\epsilon_{i},\;\mathrm{with}\;o=1,\ldots ,O-1$$

Figure 5 also presents the parameters of the ellipses. The path length $d_{i}$ between Tx and Rx is constant. *a* being the semi-major axis of the ellipse, *b* is the semi-minor axis *b* and is calculated as $b=\sqrt{a^{2}-{\left(\frac{d_{i}}{2}\right)}^{2}}$.

The UWB CIR h(t) represents the received signal over time. Every peak in the CIR is a potential person; the higher the peak, the more probable the location of the person is. To localize the person, we draw the peaks onto the target area. The position of Tx, as well as Rx must be known as they are the foci of the ellipse.

**Figure 6:**
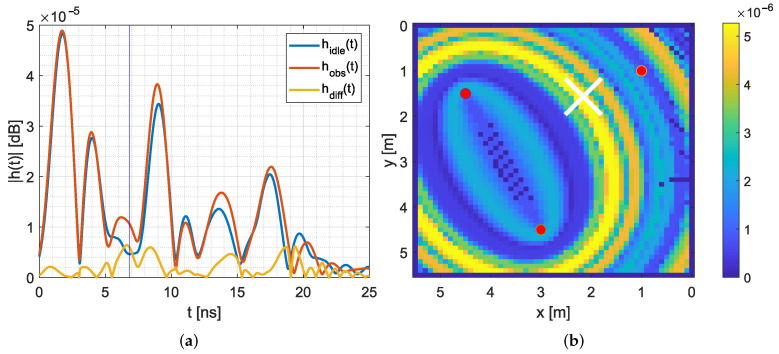
Multi-static radar principle. (**a**) Typical CIR and differences. (**b**) Heat map of MSR for a single sensor pair.

Figure 6 provides an example. Figure 6a depicts the measured and pre-processed CIR for the idle case $h_{\mathrm{idle}}\left(t\right)$ (blue CIR) and when a person is located in the target area $h_{\mathrm{obs}}\left(t\right)$ (red CIR). The blue vertical line at 7 ns represents the expected time delay $t_{\mathrm{fpP}}$ due to the position of the person. ${\mathrm{MPC}}_{0}$, the direct path, is located at 2 ns. As the direct path was not blocked by the person, this is the highest peak. The MPCs are at 9 ns and at 17 ns. Around 7 ns, we expect changes due to the position of the person, and indeed, the CIR differs from the idle case.

We calculate the difference $h_{\mathrm{diff}}\left(t\right)$ between the observed CIR $h_{\mathrm{obs}}\left(t\right)$ and the idle CIR $h_{\mathrm{idle}}\left(t\right)$, shown as the yellow line in Figure 6a. The highest differences $h_{\mathrm{diff}}\left(t\right)$ are at 7 ns and at 8.5 ns, so very close to the time delay of the person. The CIR difference is now mapped as a heat map onto the target area (see Figure 6b). Starting at the peak of ${\mathrm{MPC}}_{0}$, we draw an ellipse onto the target area with the height of the corresponding CIR difference. The white cross in Figure 6b indicates the position of the person; it lies near the perimeter where the maximum of the CIR difference is located.

To map the values of the CIR to the target area, each ellipse must be rotated and calculated [39]. For this, we calculate the center of the ellipse $m_{o}$ with:

(14) $$\left[\begin{array}{c} m_{x,o}\\ m_{y,o} \end{array}\right]=\left[\begin{array}{c} x_{Tx}+\frac{x_{Rx}-x_{Tx}}{2}\\ y_{Tx}+\frac{y_{Rx}-y_{Tx}}{2} \end{array}\right]$$

Next, we need the rotation of the ellipse:

(15) $$\alpha_{o}=\arctan\left(\frac{y_{Rx}-y_{Tx}}{x_{Rx}-x_{Tx}}\right)$$

Based on these values, we determine the radius of the ellipse within φ∈[0,2π):

(16) $$\left[\begin{array}{c} E_{x}\\ E_{y} \end{array}\right]=\left[\begin{array}{c} m_{x,o}+a\cos\left(\varphi \right)\cos\left(\alpha_{o}\right)-b\sin\left(\varphi \right)\sin\left(\alpha_{o}\right)\\ m_{y,o}+a\cos\left(\varphi \right)\sin\left(\alpha_{o}\right)+b\sin\left(\varphi \right)\sin\left(\alpha_{o}\right) \end{array}\right]$$

#### 3.3.2. General Approach

In the following, we describe how we map the ellipses for each sensor pair *o* onto the target area. For each sensor pair, one sensor will act as the transmitter and the other as the receiver.

Similar to MA-RTI, we divide the target area *A* into *J* equally sized pixels. Then, we create a heat map $v_{\mathrm{radar},o}$ for every sensor pair *o*.

Algorithm 1 shows the pseudo-code that calculates the heat map $v_{\mathrm{radar},o}$ for every sensor pair *o*. The idea of applying MSR is to extract additional changes within the CIR coming from persons. To detect changes in the CIR coming from the person, we perform so-called background subtraction as suggested by [46]. Therefore, for every sensor pair *o*, we calculate the difference:

(17) $$h_{\mathrm{diff}},o\left(t\right)=\left|h_{\mathrm{obs},o}\left(t\right)-h_{\mathrm{idle},o}\left(t\right)\right|,$$

where $h_{\mathrm{obs},o}\left(t\right)$ is the current observation and $h_{\mathrm{idle},o}\left(t\right)$ the idle CIR measurement while the target area was vacant.

The background subtraction focuses on the difference between the idle and the measured CIR measurement. By analyzing the difference, the influenced MPCs are detected. However, signal echoes on paths with a similar transmission delay $\tau_{i}$ interfere and, finally, superpose in the CIR measurement $h_{\mathrm{idle},o}\left(t\right)$. Therefore, in the case of signal echo interference, the analysis will lead to ambiguous results, reducing the localization performance of the method.   
**Algorithm 1:** Pseudo-code for multi-static radar heat map calculation adapted from [39].
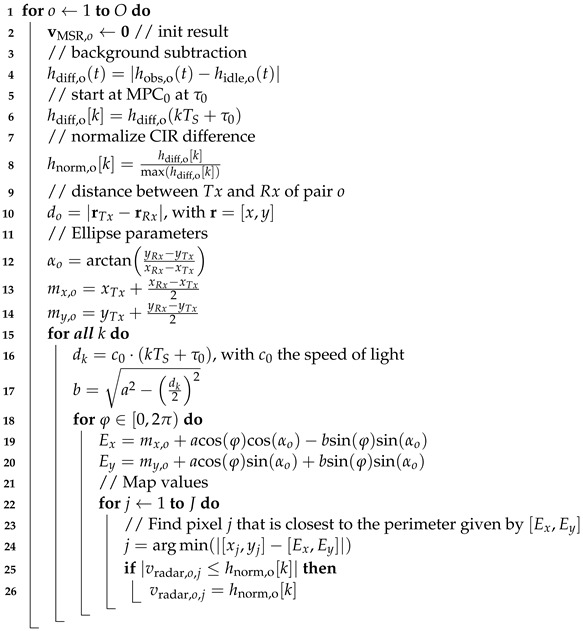


In [45], we discussed an algorithmic approach to detect and identify also interfering signal echoes successively up to a certain transmission delay difference $\Delta \tau =\tau_{i}-\tau_{j\neq i}$. However, since the placement of the anchor nodes was freely chosen in our setup, later on, we focus on anchor positions, where all transmission delays of the MPCs are distinct. Therefore, we can neglect the influence of interference in this case.

We start the mapping of the CIR difference at the peak of ${\mathrm{MPC}}_{0}$:

(18) $$h_{\mathrm{diff}},o\left[k\right]=h_{\mathrm{diff}},o\left(kT_{S}+\tau_{0}\right).$$

After background subtraction, we normalize the CIR difference:

(19) $$h_{\mathrm{norm}},o\left[k\right]=\frac{h_{\mathrm{diff}},o\left[k\right]}{\max(h_{\mathrm{diff}},o\left[k\right])}.$$

This implies that every sensor pair has the same weight.

As described in Algorithm 1, starting from the peak of ${\mathrm{MPC}}_{0}$, we calculate the ellipses with steps of k=10 according to (16), meaning that we calculate one ellipse for every 1 ns in the CIR and map them to the target area. The mapping works as follows: For every $E_{x}$ and $E_{y}$, we find the closest pixel and assign the value of the normalized CIR difference, when the current normalized CIR difference is larger than the previously saved one. Figure 6b shows the result for an ellipse that is mapped to the target area.

Finally, we calculate the mean of the heat maps:

(20) $${\hat{v}}_{\mathrm{MSR}}=\frac{1}{O}\sum_{o=1}^{O}v_{\mathrm{MSR},o}.$$

To localize the person, we determine the maximum:

(21) $${\hat{r}}_{P,\mathrm{MSR}}=\mathrm{Pos}\left(\underset{j\in \{0\ldots J-1\}}{arg max}{\hat{v}}_{\mathrm{MSR},j}\right).$$

### 3.4. Combination of Both DFL Methods

One contribution of this paper is the combination of MA-RTI and MSR. We expect that MA-RTI performs well on positions that are covered by multiple MPCs. In contrast, we expect that MSR performs well on positions that are uncovered by MPCs. In such cases, the background subtraction will be effective, and a person affects the measurements. As both methods can be applied to the same UWB CIR measurement, we expect that the combination of both methods increases the localization performance.

Both DFL methods create a heat map. MA-RTI results in ${\hat{v}}_{\mathrm{MA}-\mathrm{RTI}}$ (see (9)); MSR results in ${\hat{v}}_{\mathrm{MSR}}$ (see (20)).

As ${\hat{v}}_{\mathrm{MA}-\mathrm{RTI}}$ and ${\hat{v}}_{\mathrm{MSR}}$ are normalized, we calculate the combination of both approaches by pixelwise multiplication:

(22) $${\hat{v}}_{\mathrm{comb}}=\mathrm{diag}\left({\hat{v}}_{\mathrm{MA}-\mathrm{RTI}}\cdot {\hat{v}}_{\mathrm{MSR}}^{T}\right)$$

${\hat{v}}_{\mathrm{comb}}$ is the same size as its predecessors. As for MA-RTI and MSR, we determine the most likely position by searching the maximum

(23) $${\hat{r}}_{P,\mathrm{comb}}=\mathrm{Pos}\left(\underset{j\in \{0\ldots J-1\}}{arg max}{\hat{v}}_{\mathrm{comb},j}\right).$$

## 4. Implementation

In this section, we provide implementation details on the test setup including the hardware and software considerations. In the first part of this section, we showcase the hardware that runs the UWB radio chip, the Decawave DW1000. In the second part of this section, we introduce our firmware, which creates and extracts the CIR measurements on the sensor nodes. In the third part of this section, we present the pre-processing that is applied to the UWB CIR measurements and demonstrate how the MPCs are extracted from the UWB radio chip. In the fourth part, we provide an overview of the measurement setup that was used for the evaluation of the different DFL methods. Finally, we conclude this section by describing the flow chart of the algorithms. We provide the measurement data in the Supplementary Materials.

### 4.1. Hardware and Radio-Frequency Settings

**Figure 7:**
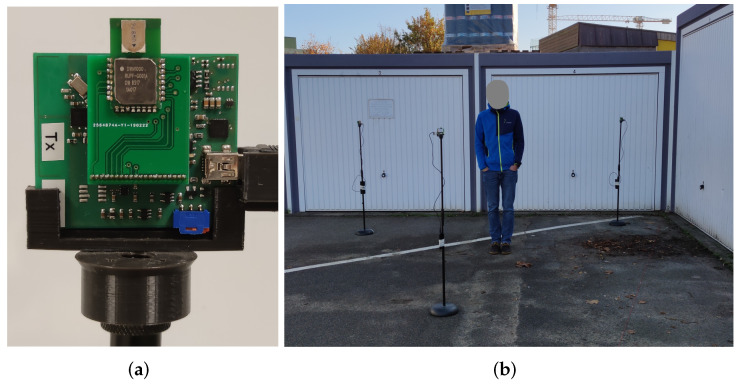
Sensor node and the test setup. (**a**) TinyTriSOS as our measurement equipment [31]. (**b**) Measurement setup in an outside environment.

In this subsection, we depict the measurement hardware and the chosen RF settings of the Decawave DW1000 radio chip. Figure 7a shows a sensor node, which is our TinyTriSOS platform utilizing a DW1000 UWB radio chip, which is controlled by an ATXMega128A1. During the measurements, the sensor nodes are powered via USB while mounted on a microphone stand at a 1.5 m height. Table 1 provides the setting of the UWB radio chip.

### 4.2. Firmware

The firmware controls the sensor nodes to exchange data via the UWB radio. Upon reception of an UWB message, the CIR and other relevant parameters are saved and transported via UWB in the next cycle. The goal of the firmware is that one node that acts as the listener listens to the communication that saves and transports the CIR payload. Next to the CIR measurement, the CIR payload contains assisting parameters that are reported by the Decawave DW1000. The listener is connected via a serial interface to a computer that logs the incoming messages.

Table 2 provides the information of the payload each node transmits. The CIR is the most important variable; for each CIR sample, we save the real and imaginary parts as a uint16_t. Currently, we measure 60 CIR samples, resulting in 60samples·2·2Byte=240 Byte as a payload. A signal with the speed of light $c_{0}$ travels approximately 0.3 m/s. Therefore, 60 ns of the CIR covers MPCs up to a distance of approximately 18 m. As an IEEE 802.15.4 frame has a maximum of 127 Byte, we had to enable extended length data frames, which enabled us to transmit frames up to 1023 Byte [47]. With the current version of the firmware, we were able to transfer CIRs measurements at approximately 20 Hz.

### 4.3. Pre-Processing of CIR Measurements

After measuring the CIR, we performed the pre-processing that we proposed in [31]. The goal of the pre-processing is to align the CIRs in time and to increase the resolution by suitable sinc-interpolation. In the following, we briefly summarize the pre-processing of the CIRs.

The measured CIR $h_{\mathrm{raw}}\left(kT_{s_{1}}\right)$ is a series of *K* I/Q values with k=0,1,…,K−1. The bandwidth of our chosen IEEE 802.15.4.a channel is B=499.2 MHz, resulting in $T_{s_{1}}\approx 1$ ns. In the firmware, we decided to set K=60 samples, which allowed us to capture a CIR with a maximum length of approximately 60 ns.

We resampled the CIR with $T_{s_{2}}$ and applied sinc-interpolation, resulting in $h_{\mathrm{IP}}\left(nT_{s_{2}}\right)$ (n=0,1,2,…), to increase the resolution. After interpolation, we aligned the CIRs in time. For time alignment, we used the timestamp from the leading edge detection of the Decawave DW1000. This timestamp is based on an integer value that has an accuracy of about 1 ns and a fine-grained fractional part that provides the result of the leading edge detection below 1 ns. We recorded and transmitted the CIR measurements of five samples prior to the reported integer part. After time alignment, we scaled each CIR with its reported $P_{\mathrm{Rxlvl}}$, which is given by (25). Finally, we cropped each CIR to be exactly *N* samples long. The resulting CIR is $h(kT_{s_{2}})$, with n=0,1,…,N−1. For the detailed information and example source code for the pre-processing, we refer to [31]. There, the alignment of the CIR in time and sinc-interpolation is explained in more detail.

### 4.4. Extraction of MPCs from UWB CIR Measurements

This section describes how the MPC values are extracted from the pre-processed UWB CIR. After extraction, the values are used for MA-RTI and MSR.

Equation (24) calculates the magnitude of the MPC in dBm:

(24) $$P_{\mathrm{MPC}}=10\;\log_{10}\left(\frac{F_{-1}^{2}+F_{0}^{2}+F_{1}^{2}}{N_{\mathrm{cnt}}^{2}}\right)-A_{\mathrm{PRF}},$$

where $F_{-1}$, $F_{0}$, and $F_{1}$ are the magnitude values of the CIR at time $\left\{t_{\mathrm{MPC}}-1\;\mathrm{ns},t_{\mathrm{MPC}},t_{\mathrm{MPC}}+1\;\mathrm{ns}\right\}$. $N_{\mathrm{cnt}}$ is the preamble accumulation count, and the constant $A_{\mathrm{PRF}}=121.74$ dB is valid for the chosen pulse repetition frequency of 64 MHz [47].

After calculating the magnitude of each MPC, we scaled $P_{\mathrm{MPC}}$ with the reported receive power level $P_{\mathrm{Rxlvl}}$ of the measured CIR. Based on [47], $P_{\mathrm{Rxlvl}}$ in dBm is calculated as

(25) $$P_{\mathrm{Rxlvl}}=10\;\log_{10}\left(\frac{C\cdot 2^{17}}{N_{\mathrm{cnt}}^{2}}\right)-A_{\mathrm{PRF}}.$$

For MA-RTI, we determine the input vector z as follows:

(26) $$\begin{array}{cc} z[i]= & z_{i} \end{array}$$

(27) $$\begin{array}{cc} \;= & |P_{\mathrm{MPC},i,\mathrm{obs}}-P_{\mathrm{MPC},i,\mathrm{idle}}| \end{array}$$

For this, we extracted for the idle reference CIR and the observation CIR the power of each MPC $P_{\mathrm{MPC}}$ with (24).

### 4.5. Measurement Setup

In the following, we outline the details of our measurement setup.

**Figure 8:**
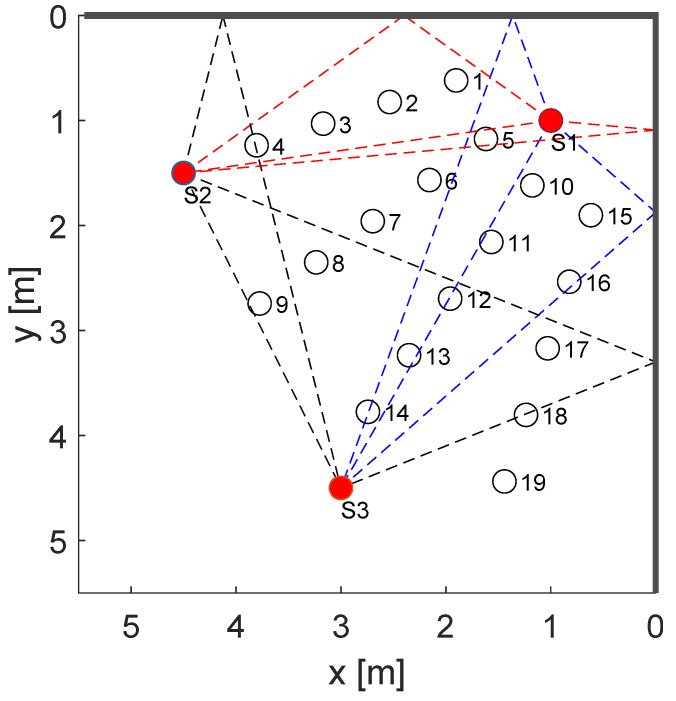
Sensor and measurements positions with signals paths of the MPCs up to the 1st order.

We measured in an outdoor scenario, with two walls (garage doors) that create strong reflections. Figure 7b shows an photograph of the setup, Figure 8 depicts the sensors and measurement positions together with the signal paths. We decided on this setup because it creates sufficient MPCs to compare the different DFL methods, without too many parasitic effects, like in an indoor environment. Still, measuring an outdoor environment creates more MPCs than measurements within an anechoic chamber [39].

The three sensor nodes were mounted on microphone stands at a 1.5 m height (depicted as red dots in Figure 8). Sensor 1 was placed at the (*x*,*y*)-coordinate (1 m,1 m), Sensor 2 at (4.5 m,1.5 m), and Sensor 3 at (3, 4.5 m), respectively. The target area spans 5.5 m in the x- and y-direction. Based on the position of the sensors and the walls, we determined the time delays for the sensor pair (1,2) with $\tau_{1}=2.56$ ns, $\tau_{2}=3.83$ ns, after $\tau_{0}$. For sensor pair (1,3), we found $\tau_{1}=4.28$ ns and $\tau_{2}=6.08$ ns after $\tau_{0}$, and for sensor pair (2,3), the delays were $\tau_{1}=9.45$ ns and $\tau_{2}=15.72$ ns.

**Figure 9:**
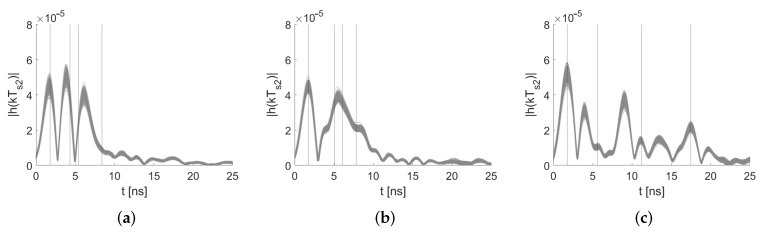
Pre-processed CIR measurements in idle target area. (**a**) Idle CIR measurements of sensor pair (1,2). (**b**) Idle CIR measurements of sensor pair (1,3). (**c**) Idle CIR measurements of sensor pair (2,3).

Figure 9 shows the pre-processed idle measurements of the three distinct node pairs. The determined first-order MPCs are depicted with solid lines. Due to superposition, the signal echoes interfere constructively and destructively and lead to the vanishing of the expected maxima. We took a closer look at this behavior in [45]. The first line indicates the peak of ${\mathrm{MPC}}_{0}$ at $\tau_{0}$; the other peaks are the theoretical positions of the MPCs. Due to constructive and destructive interference and inaccuracies in the signal paths, the position of the MPCs does not always match the maximum in the CIRs. Still, those values are required for the extraction of the MPCs for MA-RTI. In the following, we briefly discuss the effect of the misalignment of the theoretical MPCs for MA-RTI and MSR: For MA-RTI, we extract the complex values of the CIR at the respective misaligned MPCs delay. This is sup-optimal, however; a person in the proximity of an MPC will also affect the CIR in direct proximity. For MSR, the misalignment between the theoretical positions of the MPCs and the measured ones is neglectable, as we used idle measurement (with the same misalignment) for background subtraction. Note: especially at peaks in close proximity, we expect that there is no misaligned, but superposition of MPCs. To detect this, we will apply an algorithmic approach in the future; however, this is out of the scope of this paper [45].

Next to the idle measurements, we recorded at each of the 19 different reference positions CIR measurements while a person with a height of 1.95 m, a shoulder-to-shoulder length of approximately 0.45 m, and back-to-chest length of approximately 0.25 m was standing at each position. During the measurements, the person stood still, the body facing towards the south of the measurement setup. Outside of the setup, the listener node receives the UWB transmissions and forwards the data via a serial interface to a laptop, which saves the data.

Figure 8 summarizes the measurement setup with its three sensors (red dots) and 19 reference positions (black dots), as well as the signal paths up to the first order (dashed lines) between the sensor nodes. This set of reference positions enabled us to examine the advantages and disadvantages of the proposed DFL methods. Note: typically, RTI systems place sensors in a way that many signal paths cross the target area. Positions without crossing signal paths are then neglected. In contrast, MSR requires that the target area be far away from the signal paths, so that the person creates additional changes in the CIR that can be detected and processed. In the following, we describe the characteristics of the positions that are a mixture of both cases.

The measurement setup provides reference positions that cover both cases. The different positions are motivated as follows. Position 2 is approximately 0.5 m away from signal paths; therefore, we expect that this is ideal for localization with MSR. Many reference positions cross the direct path between the sensors, therefore, enabling us to determine the effect on blocked paths. The first three positions are outside the area that the direct paths of the sensors span, e.g., position 2 and position 3 are not crossed by signal paths. The fourth position is in direct proximity to the direct path and the echo path of the sensor pair (1,2) and additionally crosses the echo path of the sensor pair (2,3). Positions 6–8 are in the middle of the target area, resulting in that they are not in the proximity of the direct paths and cross only a few echo paths. We expect that MSR will perform well in those positions. Position 10–14 are placed close to the direct path of the sensor pair (1,3), and we expect MA-RTI to localize the person in those positions. Positions 15–19 are outside the middle of the target area, and only positions 16 and 18 cross an echo path. We provide the measurement data in the Supplementary Material.

### 4.6. Algorithms

**Figure 10:**
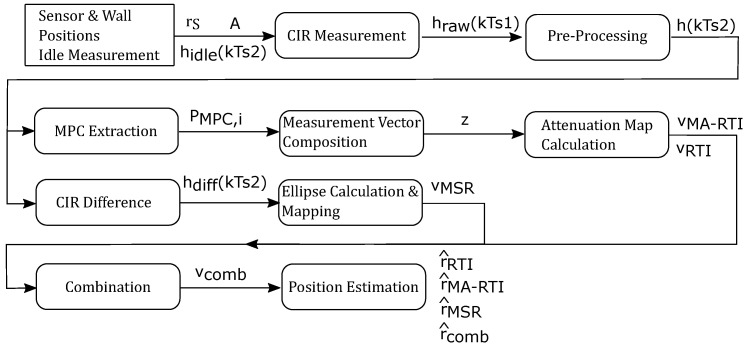
Flow chart of the proposed algorithms.

Figure 10 shows the flow chart of the algorithms we used in this paper. The initialization block requires information about the target area *A*. That includes the physical sensor $r_{S}$ and wall positions; furthermore, we recorded and pre-processed an idle UWB CIR measurement $h_{\mathrm{idle}}\left(nT_{s_{2}}\right)$, which is required for determining the absolute change of each MPC for MA-RTI and background subtraction for MSR.

For every position, or later in an online system, we acquire a CIR measurement $h_{\mathrm{raw}}\left(kT_{s_{1}}\right)$, which is then pre-processed to $h(nT_{s_{2}})$, i.e., resampled, interpolated, and time aligned. After obtaining the observation, we calculate the results of the DFL methods. For MA-RTI, we extract the power magnitudes of the MPCs $P_{\mathrm{MPC}}$ and compose the measurement vector *z*. After that, we calculate the heat map to obtain $v_{\mathrm{MA}-\mathrm{RTI}}$. For MSR, we perform the background subtraction $h_{\mathrm{diff}}\left(nT_{s_{2}}\right)$ and calculate the ellipses and map them unto the target area to obtain $v_{\mathrm{MSR}}$. Then, we calculate the combination of both methods $v_{\mathrm{comb}}$. Finally, for each method, we obtain the most likely position and determine the localization error, which is defined in the upcoming section.

## 5. Evaluation

After introducing the evaluation metrics, we present the results for the different DFL methods in this section.

### 5.1. Evaluation Metrics

We compared the different DFL methods by calculating the localization error, which is the Euclidean distance between the ground truth position and the estimated position of the chosen DFL method.

(28) $$e=\left|\right|{\hat{r}}_{p}-r_{p}\left|\right|,$$

where ||·|| depicts the Euclidean distance, ${\hat{r}}_{p}$ is the estimated position, and $r_{p}$ is the ground truth position of the person.

For every position within the target area, we calculated the mean CIR ${\bar{h}}_{\mathrm{obs}}\left(t\right)$ for each sensor pair based on 100 measurements. In addition, we determined a reference idle value $h_{\mathrm{ref}}\left(t\right)$ from the 1000 idle measurements that has its $P_{\mathrm{Rxlvl}}$ close to the mean of all $P_{\mathrm{Rxlvl}}$ [32]. In the future, we will work on an online system; therefore, we will investigate the performance of single snapshot measurements and determine adequate filters for removing noise. This idle reference is used to determine the idle MPC values and to perform the background subtraction as described for MA-RTI and MSR, respectively.

### 5.2. Evaluation of MA-RTI

In the following section, we provide and discuss the results for MA-RTI in comparison to a conventional RTI that solely relies on the direct path, i.e., ${\mathrm{MPC}}_{0}$. As described in Section 4.5, we expect that MA-RTI will outperform RTI as MA-RTI utilizes more pixels of the target area that cross an echo path by exploiting the MPCs.

For RTI and MA-RTI, we used the same parameters: the pixel width of the target area is 0.1 m [13]; the width of the weighting ellipse λ=0.01 m [13]; the pixel variance $\sigma_{v}^{2}=0.5\;{\mathrm{dB}}^{2}$ [31]; the regularization parameter $\sigma_{J}^{2}=0.5\;{\mathrm{dB}}^{2}$; the pixel correlation constant $\delta_{c}=0.5$ m [13].

**Figure 11:**
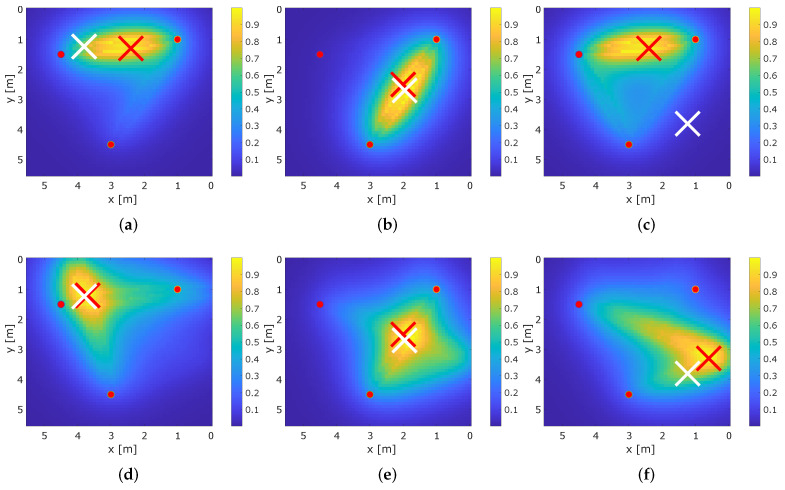
Exemplary results for RTI and MA-RTI. (**a**) RTI at position 4. (**b**) RTI at position 12. (**c**) RTI at position 18. (**d**) MA-RTI at position 4. (**e**) MA-RTI at position 12. (**f**) MA-RTI at position 18.

To demonstrate how multipath propagation improves RTI, we provide the results for the conventional RTI system that only relies on the direct path ${\mathrm{MPC}}_{0}$ and our proposed MA-RTI. Figure 11 shows exemplary the results for the RTI and MA-RTI at different positions for the same set of positions each.

The white cross indicates the ground truth position and the red cross the estimated position ${\hat{r}}_{\mathrm{RTI}}$ based on the maximum of $v_{\mathrm{RTI}}$. The values of $v_{\mathrm{RTI}}$ are color-coded in the figure, creating a heat map. Yellow color-coding means that the value is close to 1 and the probability that the person is located there is high. Dark blue color-coding means the value is close to 0 and the probability of the person’s position is low. A person that affects many signal paths is expected to create a global maximum in the heat map that is close to the ground truth position of the person.

Figure 11a,d show the results for position 4. Position 4 is in direct proximity to the direct path between (1,2) and the echo path of (1,2), as well as (2,3). For conventional RTI, the maximum value of the heat map is in the middle of the direct path between (1,2), and for MA-RTI, the maximum value of the heat map is located at the position of the person.

Figure 11b,e present the results for position 12. Position 12 is in direct proximity to the direct path of (1,3) and the echo paths of (1,3) and (2,3). As the position is in the middle of the direct path between (1,3), both RTI and MA-RTI have their maximum value of the heat map at the person’s position. As one echo path is blocked, the yellow area concentrates on the persons’ position for MA-RTI.

Figure 11c,f show the results for position 18. Here, only the echo path of (2,3) is affected; for MA-RTI, the ground-truth position of the person is approximately 1 m away from the estimated position. Still, MA-RTI estimates the position of the person at the blocked echo path, whereas the conventional RTI system provides the wrong estimate. This indicates that accurate localization requires that the person affects multiple paths.

### 5.3. Evaluation of MSR

In the following section, we provide and discuss the results for MSR.

**Figure 12:**
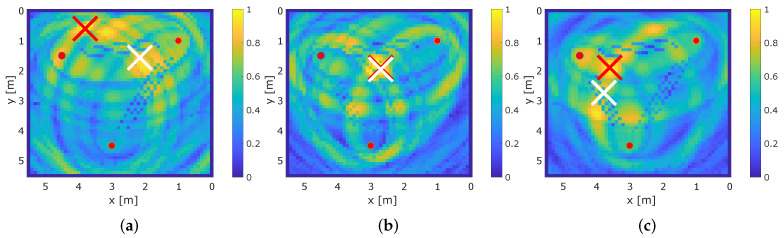
Exemplary results for multi-static radar. (**a**) MSR at position 6. (**b**) MSR at position 7. (**c**) MSR at position 9.

Figure 12 shows the results for positions 6, 7, and 9 that are within the middle of the target area. Again, the white cross indicates the ground truth position of the person and the red cross, the estimated position based on the maximum value of ${\hat{v}}_{\mathrm{MSR}}$. As only a few signal paths cross those positions, we expect good localization results on those positions.

**Figure 13:**
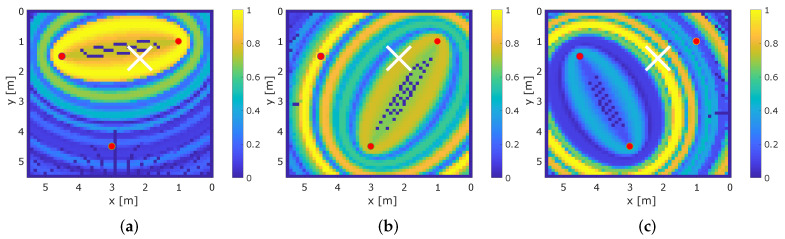
Single heat maps of sensor pairs of MSR that result in ambiguities. (**a**) MSR at position 6 pair (1,2). (**b**) MSR at position 6 pair (1,3). (**c**) MSR at position 6 pair (2,3).

Figure 12b shows the result for position 7. The global maximum of the heat map is at the ground truth position of the person. Still, there are a few local maxima that may lead to ambiguities. At position 9 (see Figure 12c), the global maximum is approximately 1 m away from the ground truth position of the person. In addition, there are other local maxima that create ambiguities. At position 6, we notice a local maximum of the heat map at the ground truth position of the person (see Figure 12a). Unfortunately, the global maximum of the heat map is located at an ambiguity at the top of the target area. To understand this behavior, we depict the results for the single sensor pairs in Figure 13.

Figure 13a shows $v_{\mathrm{MSR},o=1}$ for sensor pair (1,2). As the person is not in the proximity of a signal path, the maximum of the heat map is at an ellipse perimeter close to the position of the person. At the $v_{\mathrm{MSR},o=3}$ of sensor pair (2,3) shown in Figure 13c, the global maximum of the heat map is at the ellipse perimeter of the position of the person. The ambiguity within $v_{\mathrm{MSR},o=2}$ is caused by sensor pair (1,3), as depicted in Figure 13b. There is a global maximum of the heat map at en ellipse perimeter at the position of the person, yet the global maximum is located at an ellipse perimeter approximately 2 m further. The global maximum is caused by the person affecting an MPC, which results in a peak in the CIR difference. This global maximum results in an ambiguity when we calculate the mean of the heat maps $v_{\mathrm{MSR}}$ according to (20).

In our test setup, we recognized multiple positions where one or more of the three sensor pairs created a heat map that resulted in ambiguities.

### 5.4. Combination

In the following section, we discuss the combination of MA-RTI and MSR.

**Figure 14:**
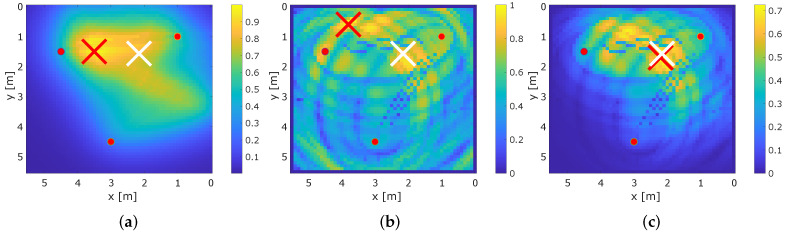
Heat maps for the MA-RTI, MSR and the combination at position 6. (**a**) Heat map of MA-RTI. (**b**) Heat map of MSR. (**c**) Heat map of the combination.

Figure 14 exemplary shows the result for all three methods for position 6. Figure 14a shows the result for MA-RTI. As the person is only in the proximity of (and not crossing) a few paths, the estimated position is approximately 1.5 m away from ground truth position of the person. The result for the multi-static radar is shown in Figure 14b. Although there is a local maximum at the ground truth position of the person, the estimated position is located at an ambiguity far away from the ground truth position. Figure 14c shows the result when both heat maps are combined. The estimated position is close to the ground truth; furthermore, ambiguities are reduced significantly.

### 5.5. Discussion

#### 5.5.1. RTI and MA-RTI

**Figure 15:**
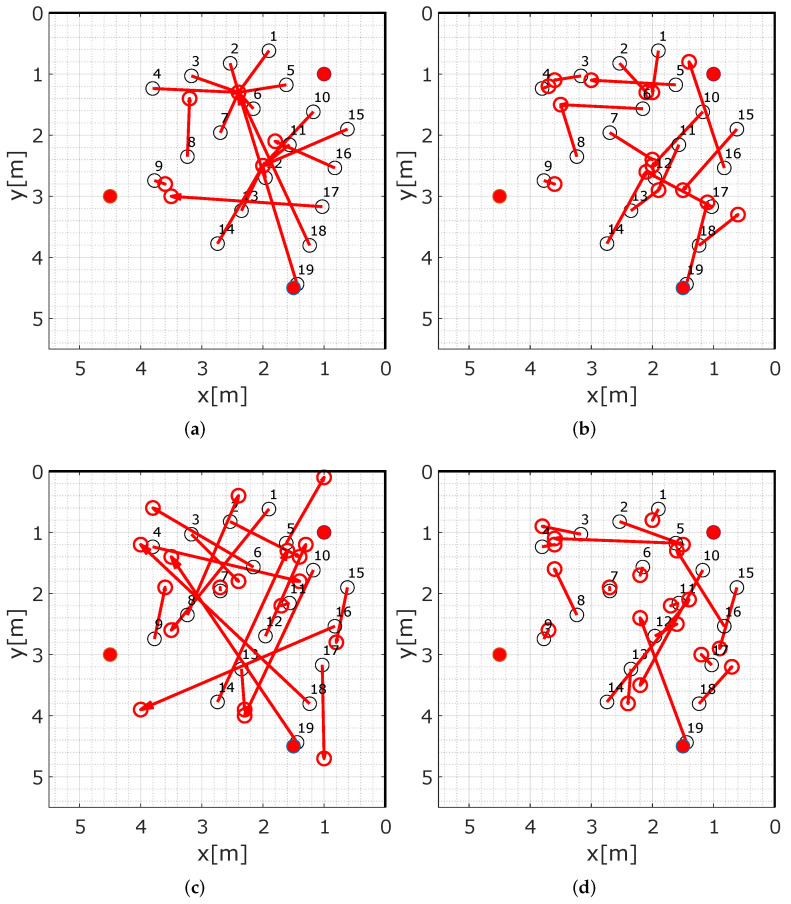
Localization results for the DFL methods. (**a**) Localization results for RTI. (**b**) Localization results for MA-RTI. (**c**) Localization results for MSR. (**d**) Localization results for the combination.

Figure 15a shows the reference positions (black circles) and the estimated positions (red circles) for conventional RTI and Figure 15b for MA-RTI. The conventional RTI is solely based on the three direct paths between the sensors. Therefore, the RTI system estimates the position of the person in the middle of the direct path (1,2) for position 1–7 and for the positions 17–19 that are outside of any direct paths. Position 9 is in the middle of the direct path of (2,3) and is estimated correctly. Positions 10–15 are a direct path between (1,3), the position estimated results in the middle of this MPC. Figure 15b shows the localization errors for MA-RTI. Whereas the RTI system results in a big cluster for positions 1–8, the localization errors of MA-RTI are overall closer to the ground truth positions. Still, position estimations for positions 10–19 are often between position 12 and 17, because different direct and echo paths overlap there.

**Figure 16:**
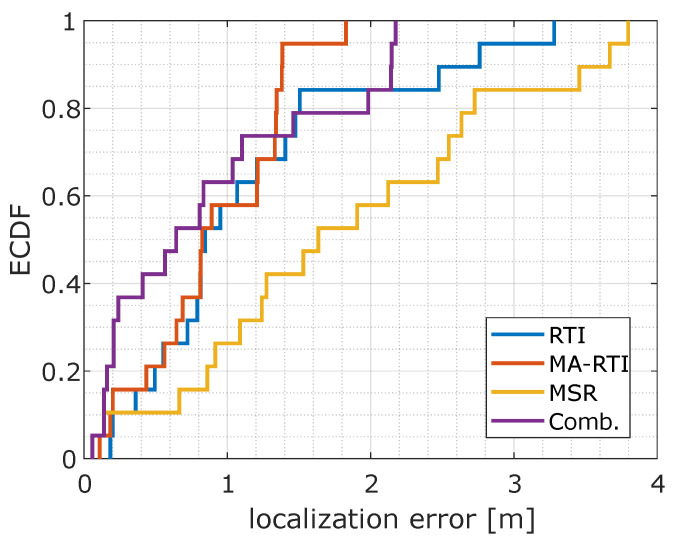
ECDFs of the localization errors.

Figure 16 shows the empirical cumulative distribution function (ECDF) for the different DFL methods. MA-RTI outperforms conventional RTI. Based on the ECDFs shown in Figure 16, we determined the following localization errors: RTI, which only exploits the direct path results in 50% of all cases in a localization error of 0.84 m and in 80% of all cases in 1.5 m. Our proposed approach MA-RTI, which includes multipath propagation, results in 50% of all cases in a localization error of 0.82 m and in 80% of all cases in 1.34 m. Furthermore, the localization results are closer to the ground truth positions. Based on the results, we concluded that multipath propagation benefits RTI algorithms. MPCs aid in covering the target area; in addition, by extraction from the UWB CIR, we combat the problem of narrowband RTI with multipath propagation. With three physical sensors, we can cover the target area with a well-known algorithm. We expect that we can increase the accuracy of MA-RTI by increasing the number of paths even further. We achieve that by increasing the number of sensors or by extracting more MPCs from the CIR. In the future, we will set up the system in an indoor environment, e.g., an office that has at least four walls, creating more signal paths, and evaluate the system’s performance in an indoor environment.

#### 5.5.2. MSR

Figure 15c visualizes the localization errors for MSR. Only a few positions (7, 9, 11, 13, 15) that are not covered by MPCs provide an accurate location estimate. We noticed many outliers that were caused by a single bad sensor pair. Based on the ECDFs shown in Figure 16, we determined the following localization errors: The multi-static CIR radar results in 50% of all cases in a localization error of 1.63 m and in 80% of all cases in 2.72 m.

#### 5.5.3. Combination

When comparing the ECDF of the combination of MA-RTI and MSR with the other DFL approaches in Figure 16, the combination provides a lower localization error than MSR and is close to MA-RTI. Figure 15d depicts the localization errors for the measurement positions. Compared to the MSR in Figure 15c, the results improved significantly. The outliers became fewer. In comparison to the results of MA-RTI, there was no clustering of the position estimates around positions 12 and 17. The combination of MA-RTI and MSR results in 50% of all cases in a localization error of 0.64 m and in 80% of all cases in 1.98 m. Therefore, the combination improves the localization for the majority of the positions.

In the future, we will work on improving the MA-RTI results and MSR and deploy and evaluate the system in an indoor environment with more MPCs. If required, we will measure with more sensors, possibly increasing the localization accuracy even more.

## 6. Conclusions and Future Work

Two promising approaches for device-free localization (DFL) have been identified in the past: radio tomographic imaging (RTI) and multi-static radar (MSR). In this paper, we show how RTI benefits from the analysis of multipath propagation and show how MSR deals with multipath. We exploit the ultra-wideband (UWB) channel impulse response (CIR) measurements with four different DFL methods. To test and compare those DFL methods, we created a test setup with three sensor nodes and measured the UWB CIR with a commercially available off-the-shelf UWB radio chip, the Decawave DW1000. With our setup and 19 reference positions, we achieved the following results: RTI, which exploits the direct path without considering multipath in the CIR, results in a localization error of better than 0.84 m in 50% of all cases and in 1.5 m in 80% of all cases. Our proposed approach considering multipath effects MA-RTI results in a localization error of 0.82 m for 50% of the cases and in an error of 1.34 m for 80% of all cases. In comparison, MSR results in a localization error of 1.63 m (50% of the cases) and in an error of 2.72 m (80% of the cases). MSR performs worse, because the background subtraction applied to the measurement results does not compensate for all multipath propagation effects. Therefore, we propose a combination of MA-RTI and MSR that results in the best performance with a localization error of 0.64 m (50% of the cases) and 1.98 m (80% of the cases).

In the future, we will enhance the system and deploy it in an office-type indoor scenario. The indoor scenario will increase the effects of multipath, which can improve the localization results, if we manage to handle the complexity of the problem. Furthermore, we will implement a live system for evaluation and demonstration.

## Figures and Tables

**Table 1:** RF settings of the Decawave DW1000 radio chip.

Option	Setting
IEEE 802.15.4.a channel	3
center frequency $f_{c}$	4.4928 GHz
bandwidth *B*	499.2 MHz
pulse repetition frequency	64
preamble length	128
preamble acquisition chunk size	8
Tx and Rx preamble code	9
data rate	6.8 MBit/s
PHY header mode	extended length data frames

**Table 2:** CIR payload.

Variable	Type	Meaning
intPart	uint16_t	integer part of CIR for time alignment
fracPart	uint16_t	fractional part of CIR for time alignment
maxNoise	uint16_t	maximum noise level of CIR
stdNoise	uint16_t	standard deviation of noise level of CIR
maxGrowthCIR	uint16_t	max. value of CIR
rxPreamCount	uint16_t	preamble count $N_{\mathrm{cnt}}$ of CIR
fppl	double	$P_{\mathrm{MPC},0}$
rxlvl	double	$P_{\mathrm{Rxlvl}}$
nodeId	uint16_t	NodeID of the CIR
CIR	uint16_t[]	CIR data

## Data Availability

We provide the measurement data in the Supplementary Material.

## Supplementary Materials

The following Supporting Information can be downloaded at: https://www.mdpi.com/article/10.3390/s22166255/s1.

## Abbreviations

The following abbreviations are used in this manuscript:

CIR	channel impulse response
COTS	commercial off-the-shelf
DFL	device-free localization
ECDF	empirical cumulative distribution function
MA	multipath-assisted
MPC	multipath component
MSR	multi-static radar
RTI	radio tomographic imaging
RF	radio frequency
RSS	received signal strength
UWB	ultra-wideband
